# Genome-wide identification of Major Intrinsic Proteins in *Glycine soja* and characterization of GmTIP2;1 function under salt and water stress

**DOI:** 10.1038/s41598-017-04253-z

**Published:** 2017-06-23

**Authors:** Da-yong Zhang, Manoj Kumar, Ling Xu, Qun Wan, Yi-hong Huang, Zhao-Long Xu, Xiao-Lan He, Jin-Biao Ma, Girdhar K. Pandey, Hong-Bo Shao

**Affiliations:** 1 Salt-soil Agricultural Center, Institute of Agricultural Resources and Environment, Jiangsu Academy of Agricultural Sciences, Zhongling Street No.50, Nanjing, 210014 China; 2 Department of Plant Molecular Biology, University of Delhi South Campus, Benito Juarez Road, Dhaula Kuan, New Delhi, 110021 India; 3 Key Laboratory of Biogeography and Bioresources in Arid Land, Xinjiang Institute of Ecology and Geography, Chinese Academy of Sciences Urumqi, Urumqi, China; 4 JLCBE, Yancheng Teachers University, Xiwang Avenue 1, Yancheng, 224002 China; 5 School of Biotechnology, Jawaharlal Nehru University, New Delhi, 110067 India

**Keywords:** Plant sciences, Plant stress responses

## Abstract

In different plant species, aquaporins (AQPs) facilitate water movement by regulating root hydraulic conductivity under diverse stress conditions such as salt and water stresses. To improve survival and yield of crop plants, a detailed understanding of stress responses is imperative and required. We used *Glycine soja* genome as a tool to study AQPs, considering it shows abundant genetic diversity and higher salt environment tolerance features and identified 62 *GsAQP* genes. Additionally, this study identifies major aquaporins responsive to salt and drought stresses in soybean and elucidates their mode of action through yeast two-hybrid assay and BiFC. Under stress condition, the expression analysis of AQPs in roots and leaves of two contrasting ecotypes of soybean revealed diverse expression patterns suggesting complex regulation at transcriptional level. Based on expression analysis, we identify *GmTIP2;1* as a potential candidate involved in salinity and drought responses. The overexpression of GmTIP2;1 in *Saccharomyces cerevisiae* as well as *in-planta* enhanced salt and drought tolerance. We identified that GmTIP2;1 forms homodimers as well as interacts with GmTIP1;7 and GmTIP1;8. This study augments our knowledge of stress responsive pathways and also establishes GmTIP2;1 as a new stress responsive gene in imparting salt stress tolerance in soybean.

## Introduction

Being sessile, plants encounter numerous stimuli including favorable and non-favorable conditions in their typical growth habitat. This is a challenging task specifically under constantly changing climate conditions; plants do depend on proper water transport mechanism to cope well against these adverse conditions^1^. Plants absorb water through roots and transport it to the rest of the aerial parts, which is broadly mediated through plant water channel known as aquaporins. Aquaporins (AQPs) are an ancient family of channel proteins that transport water and other solutes through pores in the cell membranes, and these are found in all eukaryotes and most prokaryotes^2^.

In higher plants, AQPs show certain sequence conservation with putative membrane spanning domain and two highly conserved Asn-Pro-Ala (NPA) motifs^3^. Studies on the detailed function of AQPs in plants have been limited due to larger morphological and genetic diversity amongst different species along with challenging methodologies for measuring water transport. However, in recent years, since the development of multiple model plant species with available genome sequences, various evidences have suggested that AQPs significantly contribute to water transport in plants through roots^4, 5^. The plant AQPs have been reported playing important roles in soil-water relations, cell osmoregulation, seed ripening, drought and salt tolerance^6–12^. Thus, it is important to study and identify AQPs responding differentially towards environmental cues and study their potential role under different stress conditions, which can potentially provide novel candidates for genetic engineering strategy for improving crop quantity and quality in the future.

In this regard, we chose to study AQPs in soybean both cultivated (*Glycine max*), which is one of the important agricultural crops and is categorized as a moderately salt-sensitive, and wild-soybean (*Glycine soja*), which is known to be tolerant to higher salt environment. The *Glycine max* genome contains large number of AQPs (GmAQP) compared to Arabidopsis, including 22 GmPIPs, 23 GmTIPs, 13 GmNIPs, 6 GmSIPs and 2 GmXIPs, probably because of higher degree of ploidy in the genome^13, 14^. It will take long to individually characterize each of the AQPs from soybean, however recently many reports have been published describing involvements of certain AQPs from soybean in abiotic stress response, which could potentially identify important candidates to characterize *in-planta* ^15–19^. A recent study found that one of the genes of *Glycine soja*, *GsTIP2*;*1* overexpression in Arabidopsis reduces tolerance to salt and dehydration stresses^20^. Another report indicated that constitutive overexpression of a soybean plasma membrane intrinsic protein-encoding gene, *GmPIP1*;*6*, confers salt tolerance to soybean^21^. These reports imply that GmAQPs are involved in mediating stress sensitivity, however more detailed study is needed to ascertain the functional role played by specific AQPs in soybean.

In the present study, we have made an attempt to explore the role of soybean AQPs in the abiotic stress responses. Considering the fact that *Glycine soja* is relatively salt tolerant, we decided to explore its genome and identify all major intrinsic proteins and compare whether there are significant differences between GmAQPs and GsAQPs. We further evaluated the spatial and differential expression pattern of *Glycine max* AQPs using RNA-seq based expression analysis. In this study, we identified *GmTIP2;1* as a novel candidate, involved in providing salt and water stress tolerance to the plants. We also acquired deeper mechanistic insight in the role of GmTIP2;1 by identifying its interacting partners, GmTIP1;7 and GmTIP1;8, suggesting the involvement of these three AQP genes in synchrony to impart tolerance to salt and water limitation conditions in soybean.

## Results

### Identification and characteristics of *Glycine soja* AQP genes

**Figure 1:**
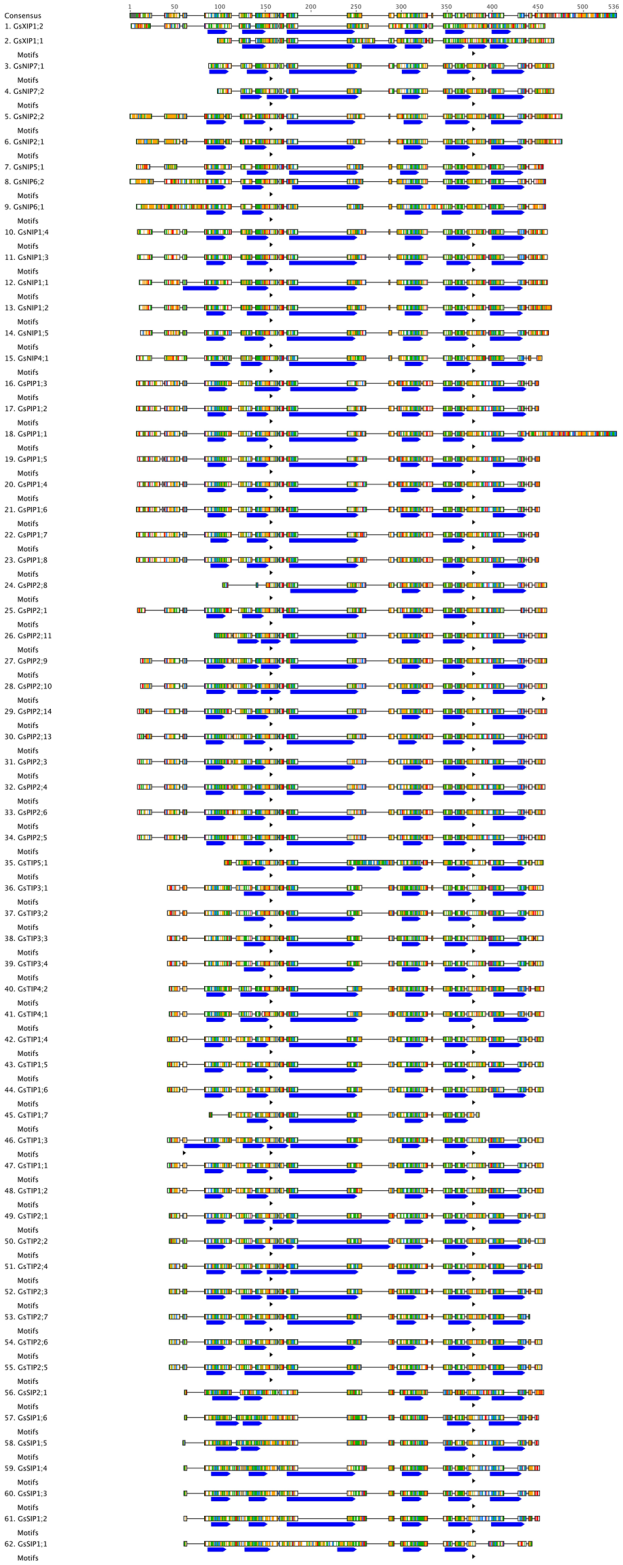
Protein sequence alignment and motif analysis of GsAQPs. Multiple sequence alignment of the full-length amino acid sequences of GsAQPs. The protein sequences were aligned using Muscle alignment tool with default parameters on Geneious 7.1 software. The similarity is depicted through colors in Clustal format. The NPA motifs (black small arrow) were identified using motif detection tool in Geneious 7.1. The transmembrane region (TMR) domains were predicted using transmembrane prediction tools in Geneious 7.1, and depicted as blue colored boxes.

To find all AQP genes in *Glycine soja*, HMM (hidden Markov model) search was performed against all predicted proteins from the *Glycine soja* genome. A total of 82 proteins were identified out of which 20 proteins were omitted because of having only 1 or 2 transmembrane domains or being the isoforms of other genes. Thus, in total 62 proteins were identified as GsAQPs and confirmed by BLASTP against GmAQPs, which is in good agreement with earlier performed studies^13^. The length of these proteins ranged from 160 to 380 amino acids. We further analysed structural similarities and differences between these GsAQPs. Multiple sequence alignments of Gs AQPs, revealed sequence homology amongst these proteins especially at their transmembrane regions (TMRs) and NPA motifs, both of which are the main characteristics of AQPs (Fig. 1). Majority of AQPs studied, contained two NPA motifs except one having three and eight having one NPA motif, suggesting their conserved biological functions. The AQPs having one NPA motif belonged to XIP and SIP subclass, which has substitution at “A” with either “T”, “L” or ‘S”. We further analyzed their location in genome and presence of number of TMR. These genes were located on different chromosomes in the soybean genome (data not shown) and had 4–7 TMR (Fig. 1 and Supplementary Table 1). Overall, we observed very high similarity amongst the AQPs between cultivated and wild-soybean.

### Tissue specific expression analysis of *GmAQP* genes based on RNA-seq data

**Figure 2:**
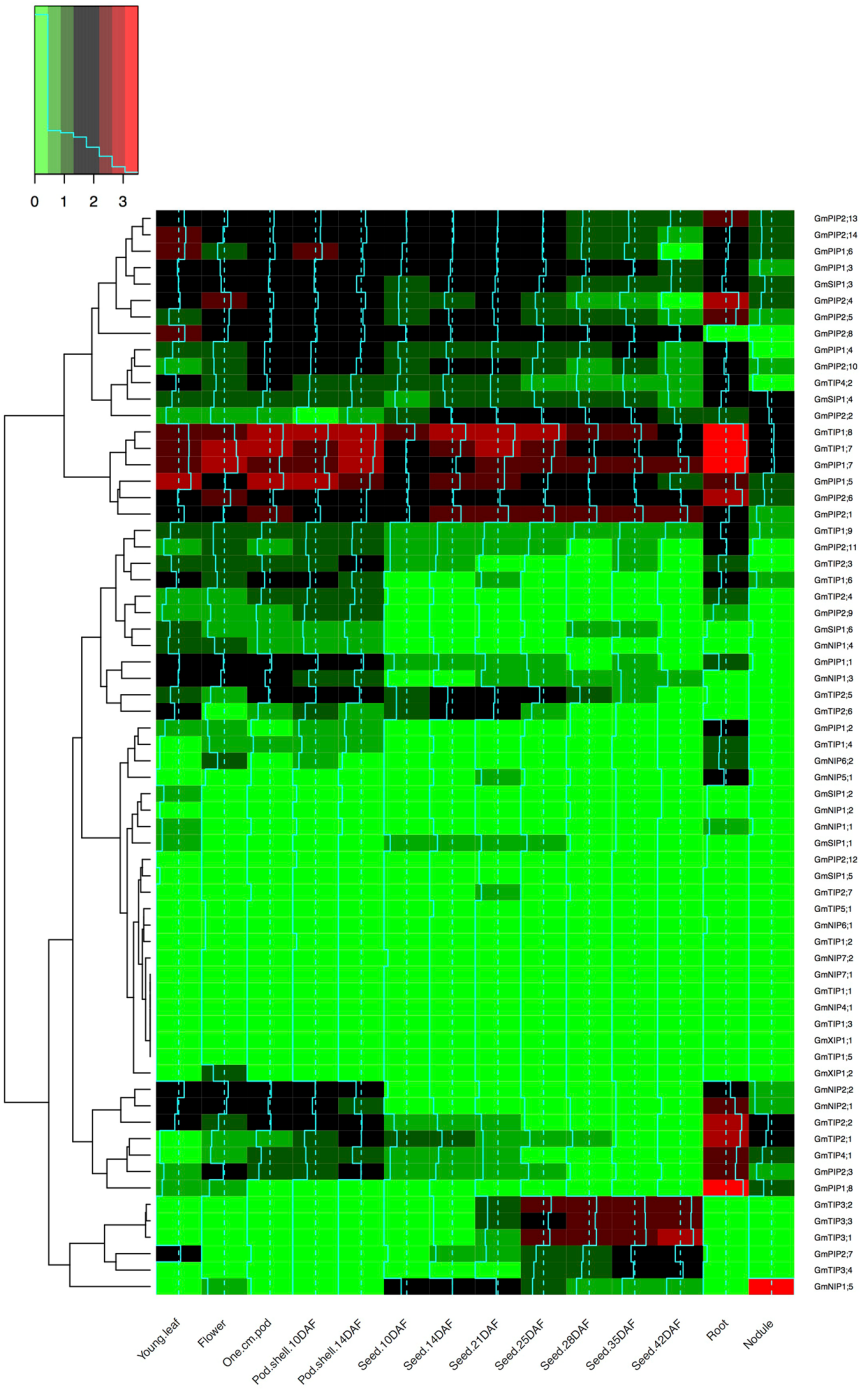
Heatmap profiles of the *GmAQPs* in tissues. Relative tissue expression levels of *GmAQPs* based on RNA-seq data were used to construct the expression patterns of soybean genes. The expression data values were plotted as heat map and clustered using R software. The color bar at the top shows the range of expression from lowest expression level (green) to highest expression level (red), black is the median expression level.

Considering that *Glycine soja* AQPs showed high degree of homology with GmAQPs, we decided to determine the expression pattern of GmAQPs in plant tissues. We acquired the *Glycine max* transcriptome data of different tissues from publically available RNA-seq datasets (http://soybase.org/soyseq/)^22^ and sorted the expression values of GmAQPs. The heatmap of expression data revealed diverse pattern of expression of *GmAQPs* in various tissues (Fig. 2). We observed two clusters with relatively higher expression in roots, and concentrated our efforts to explore them further assuming the primary role of AQPs in water transport through root. These clusters mainly contained members from PIP, TIP and NIP families. The heatmap analysis revealed that *GmTIP1*;*7*, *GmTIP1*;*8* and *GmPIP1*;*7* are most highly expressing genes in root tissues.

### Expression analysis of *GmAQP* genes in response to salt and dehydration stress

**Figure 3:**
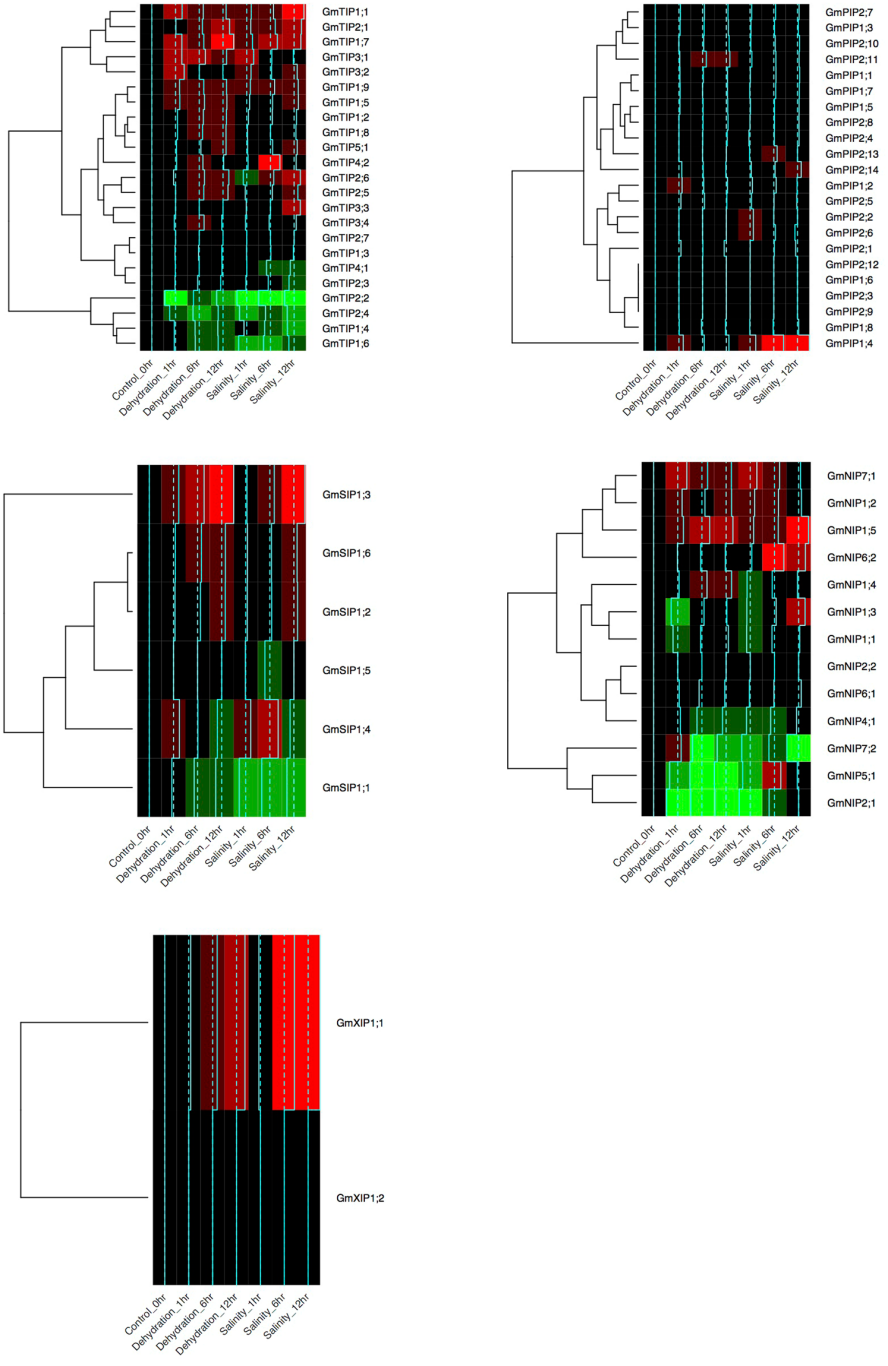
Expression profiles of *GmAQPs* under salt and dehydration. Expression of different *GmAQPs* after salinity and dehydration stress at different time points plotted as heatmaps. The expression data (Reads Per Kilobase Million) values were plotted after normalization for each gene with respect to control. The color pattern shows the range of expression from lowest expression level (green) to highest expression level (red), black is the median expression level.

To further investigate the response of *AQP* genes under abiotic stress conditions like salt and dehydration, we utilized the publically available RNA-seq data (GEO accession no. GSE57252)^23^. After processing the RNA-seq data, we fetched expression values of *GmAQP*s. The expression data was normalized and heatmap analysis and clustering was performed using R software. The heatmap was plotted separately for different classes of *AQP* genes. The *GmTIPs* analysis revealed that expression of *GmTIP2*;*1*, *GmTIP1*;*7* and *GmTIP1*;*1* increased after 12 hrs of salt and dehydration stress (Fig. 3). Interestingly, *TIP* genes showed overlapping differential response to dehydration stress and salt stress. In addition, 4 *TIPs* showed down regulation upon salt and dehydration stress. The analysis of *PIPs* showed inconsistent expression pattern in response to salt and dehydration stresses. However, one of the members, *GmPIP1*;*4* showed high expression in salt stress condition. Similarly, *GmSIP*, and *GmNIP* members also showed variable expression pattern and lacked any consistency. However, *GmXIP1*;*1* showed high expression under both salt and dehydration stress conditions compared to *GmXIP1*;*2*.

**Figure 4:**
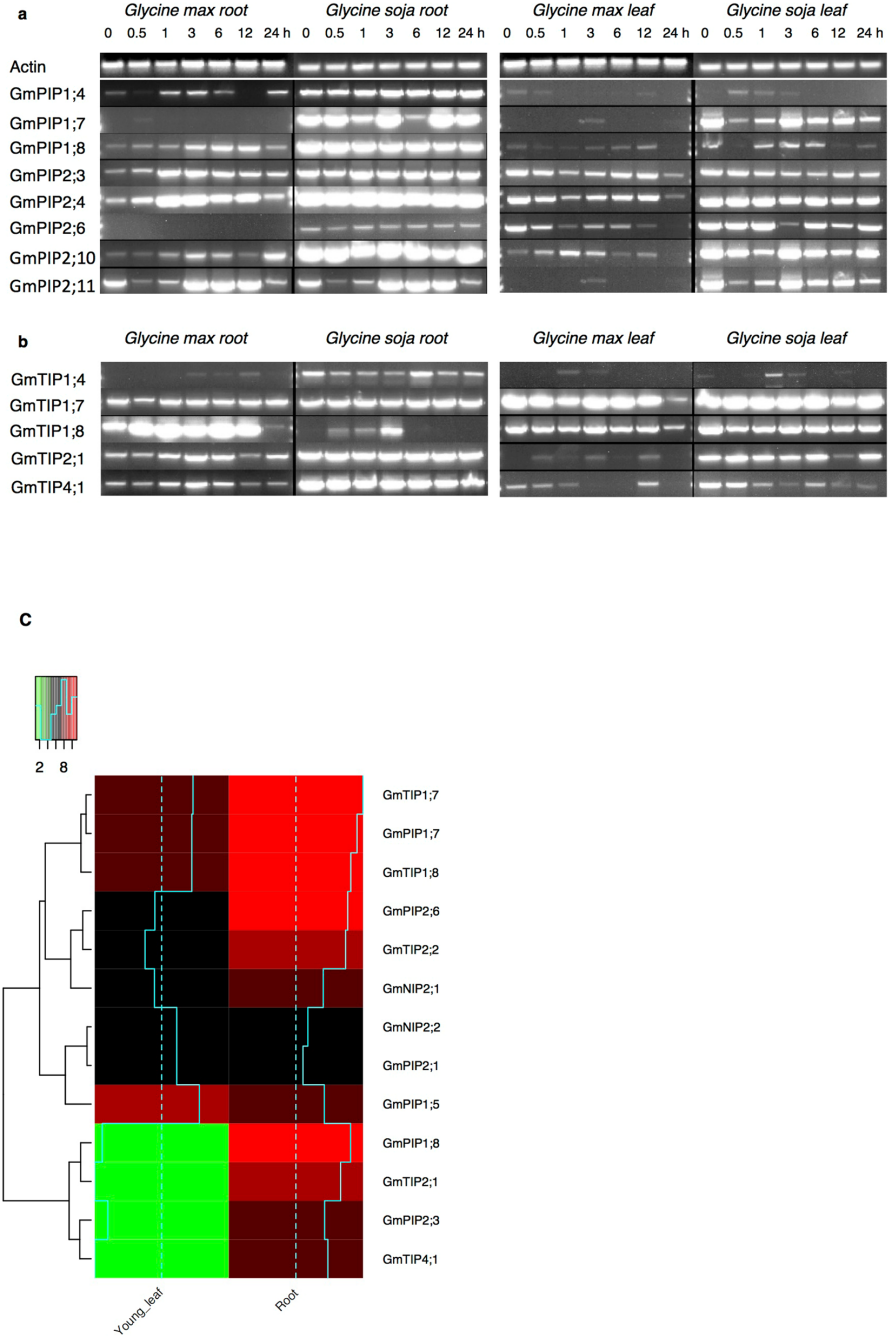
Expression analysis of *GmPIP and GmTIP* genes under salt stress in *Glycine max* and *Glycine soja*. The expression of respective genes were analysed using RT-PCR, which was performed using specific primers listed in Supplementary Table S2. The numbers denote time after salinity treatment (200 mmol L^−1^ NaCl) in hours (0 to 24 hours). Soybean *actin* gene was used as an internal control, and DNA gels were analysed under similar experimental conditions. Left panel represents gene expression analysis for root samples and right panel from leaves samples derived from *Glycine max* and *Glycine soja* genotypes. (**a**) Expression of *GmPIP* genes. (**b**) Expression of *GmTIP* genes. (**c**) Quantitative relative expression of selected *GmPIP*, *TIP* and *NIP* genes under control condition.

We further validated qualitatively the expression of selected *TIPs* and *PIPs* under salt stress condition at different time intervals in root and leaf tissues. We observed that under salt stress, expression of all the *GmPIP* genes was increased in root tissues (Fig. 4a). On the other hand, no coordinated expression of *GmPIP* genes was observed in leaves under physiological conditions. However, upon induction of salt stress, increased expression of *GmPIP* was observed (Fig. 4a).

Semi-quantitative gene analysis showed variable expression of *GmTIP* genes in roots and leaves of both genotypes. *GmTIP1*;*4* did not show any basal expression but was induced in response to salinity stress although comparatively less than other *GmTIPs* (Fig. 4b). While all *GmTIP* genes showed differential expression in root and leaves in response to salinity; *GmTIP1*;*8*, turned out to be an exception. In stark contrast to all *GmTIPs*, *GmTIP1*;*8* expression was found to be reduced after 3 hours of salt stress treatment in *Glycine soja* roots. *GmTIP2*;*1* was relatively less expressed in *Glycine max* roots compared to *Glycine soja* where its basal expression was already very high and the expression remained higher after salt stress as well. *GmTIP1*;*7* was differentially expressed in roots of two genotypes in response to salinity stress although its expression in leaves was quite uniform. Overall, we observed that most of the analysed *PIPs* and *TIPs* have higher basal expression in *Glycine soja* compared to *Glycine max* in both roots as well as leaves.

Additionally, we quantified the basal expression of selective *Glycine max* *TIP*, *PIP* and *NIP* genes between root and leaf tissues using the RNA-seq data and plotted the heatmaps, which revealed that *TIP* members *GmTIP1*;*7*, *GmTIP1*;*8* and *GmTIP2*;*1* and *PIP* members *GmPIP2*;*6* and *GmPIP1*;*8* had maximum expression in roots and *GmNIPs* were not highly differentially expressed (Fig. 4c).

Overall, these results indicate that salt stress lead to increase in expression of *GmPIPs* and *GmTIPs*, however the expression is fairly differential with respect to specific tissue and the period of stress treatment.

### Promoter sequence analysis of differentially expressed genes

Promoter *cis*-elements are known to play vital roles in regulating gene expression, thus, we performed *in-silico* analysis to identify any particular motif in the promoters of differentially expressed *GmAQPs*. We analysed the 1.5 kb upstream region of genes and found that most of these promoter sequences have several binding sites for different transcription families like, AP2, WRKY, MYB, bHLH, bZIP etc. These transcription factor families are known to be involved in regulating the stress responsive genes, however, this needs to be validated in future studies.

### Identification of protein interaction networks among selected GmAQPs

**Figure 5:**
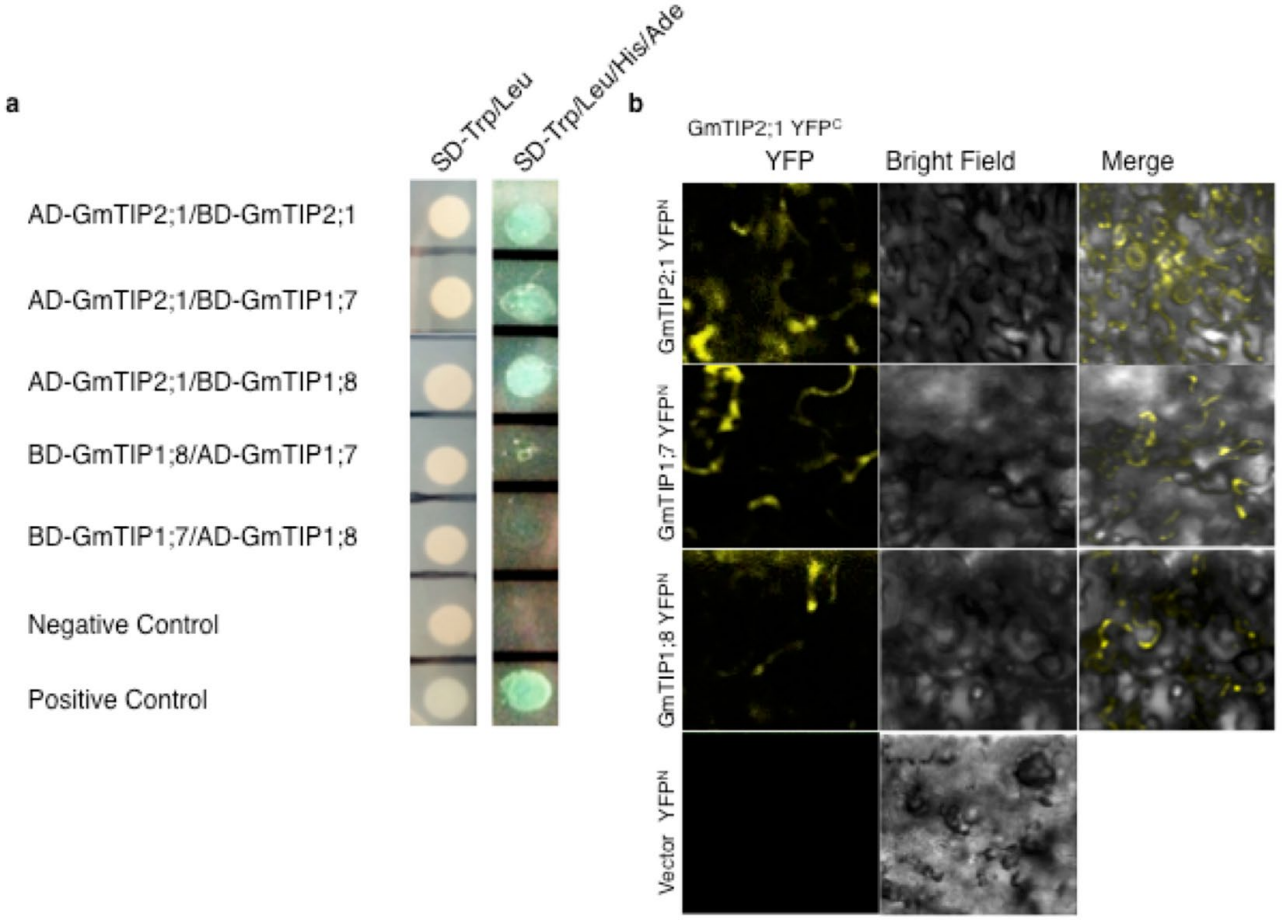
*In vivo* protein interaction between GmTIP2;1, GmTIP1;7 and GmTIP1;8. (**a**) Yeast two-hybrid assay analysis identified that GmTIP2;1 can form homodimers and heterodimers in yeast cells. The full-length ORF sequences of *GmTIP2*;*1*, *GmTIP1*;*7* and *GmTIP1*;*8* were cloned and ligated into pGADT7 and pGBKT7 vectors. After co-transformation of the baits and preys, equal amounts of yeast clones were plated on SD-Leu-Trp and SD-Leu-Trp-His-Ade + X--gal selective plates, and the plates were incubated at 30 °C until formation of the colonies. Yeast cells carrying pGBKT7–53 and pGADT7-SV40 plasmids were used as positive controls, and those with pGBKT7-Lam and pGADT7-SV40 were used as negative controls. (**b**) Bimolecular fluorescence complementation (BiFC) analysis for conducting the interaction studies in tobacco leaves. The assay was performed to confirm the results of the yeast two-hybrid assays. *GmTIP2*;*1*, was fused to the N-terminal (YN) and *GmTIP2*;*1*, *GmTIP1*;*7* and *GmTIP1*;*8* were fused to the C-terminal (YC) halves of YFP and were imaged using a confocal microscope after incubation at room temperature for over 18 h. The images are shown as YFP, merged YFP and bright field. The last panel shows the negative control.

The alignment and gene structure analysis suggested high sequence conservation and common ancestral origin amongst AQPs, suggesting these might have redundant roles and can interact amongst themselves to perform certain specific functions, we performed yeast two-hybrid experiment to examine this hypothesis. Ten differentially expressing AQPs including *GmTIP2;1*, *GmTIP1;7*, *GmTIP1;8, GmPIP2;3*, *GmPIP2;3*, *GmPIP1;4*, *GmPIP2;4*, *GmPIP1;8*, *GmPIP2;6*, *GmPIP2;10*, *GmPIP2;11* and *GmPIP1;7* were selected and screened using yeast two-hybrid assay to examine the possible interactions. The results showed that GmTIP2;1 could interact with itself as well as with GmTIP1;7 and GmTIP1;8 in yeast cells (Fig. 5a). Beside this, we did not observe any interactions between other proteins in yeast two-hybrid assay. To validate, we further examined these interactions *in planta* using a Bimolecular fluorescence complementation (BiFC) assay via transient expression in leaves of *N*. *benthamiana*. These proteins were fused with N-terminal (YN) and C-terminal (YC) halves of YFP, and the interactions depending on proximity of proteins led to reconstitution of YFP fluorescence being visualized as strong YFP signal (Fig. 5b). Taken together, these results confirmed that GmTIP2;1 can form homodimers as well as heterodimers by interacting with GmTIP1;7 and GmTIP1;8.

### Overexpression of *GmTIP2;1* improved salt and drought tolerance in *Saccharomyces cerevisiae*

**Figure 6:**
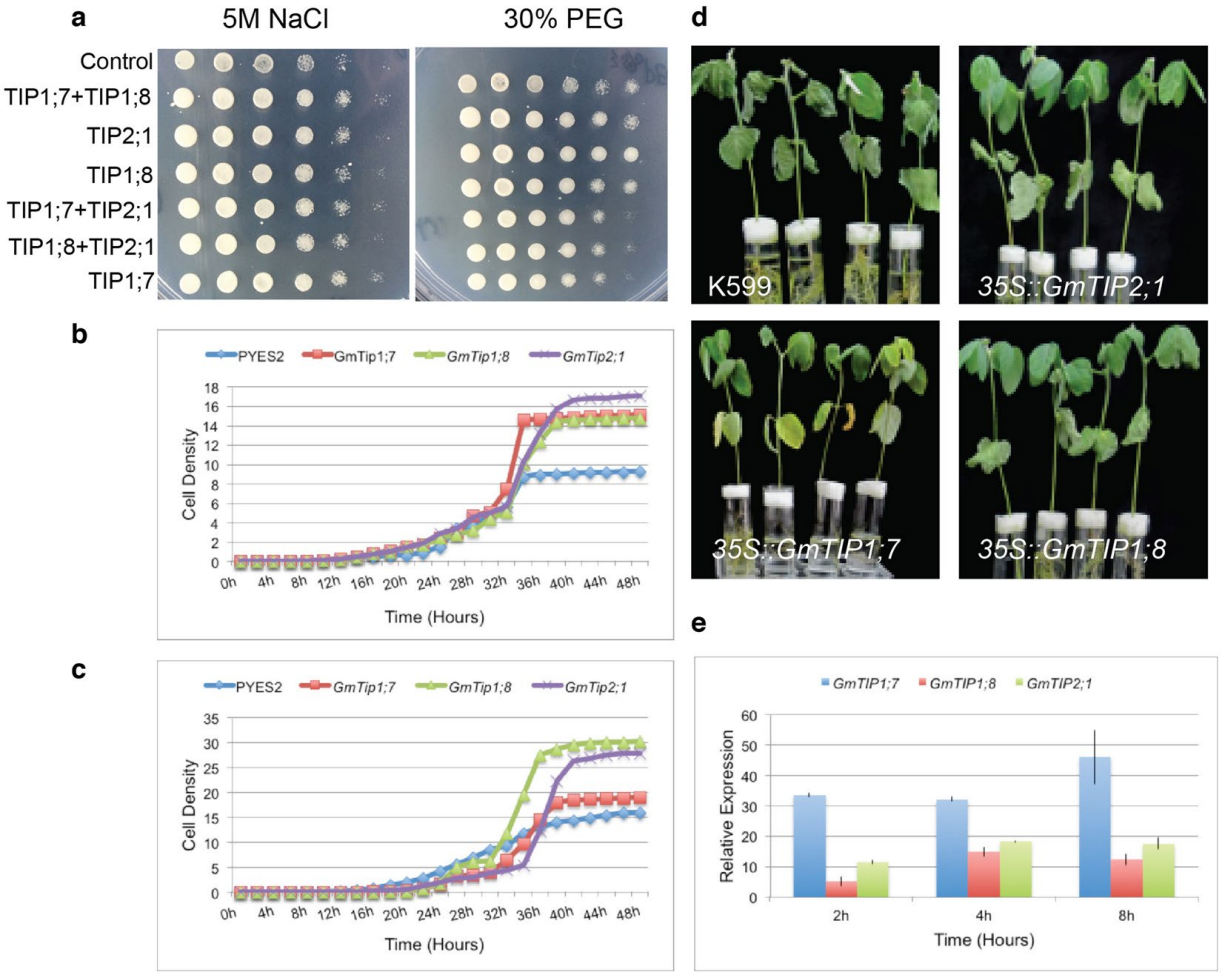
Overexpression of *GmTIP2;1*, *GmTIP1;7* and *GmTIP1;8* improved salt and drought tolerance in *Saccharomyces cerevisiae* and soybean. (**a**) Various combination of overexpressing yeast cells were plated on 30% PEG 6000 and 5 M NaCl into SC-Ura medium. According to the survival patterns of the yeast cells, it is visible that overexpression of GmTIP2;1 improved salt and drought tolerance in *Saccharomyces cerevisiae*. (**b**,**c**) Growth curves depict analysis of the growth of yeast strains overexpressing the indicated proteins in YPDA medium supplemented with NaCl (0.5 M) and PEG (10%). X-axis shows time and Y-axis shows OD600 values. OD were measured every 2 h. (**d**) Phenotypic analysis of the hairy root transformed transgenic plants treated by 200 mM NaCl solution for seven days. The photograph was documented after 7 days of growth of these respective seedlings in hydroponic media containing 200 mM NaCl. **(e)** Real-time RT-PCR analysis of *GmTIP2;1*, *GmTIP1;7* and *GmTIP1;8* under drought stress: cultivated soybean seedlings were treated with 30% PEG 6000 for 0, 2, 4 and 8 h (P0–8) and relative expression of *GmTIP2;1*, *GmTIP1;7* and *GmTIP1;8* were plotted compared to control.

To investigate the biological functions of *GmTIP2;1* *GmTIP1;7* and *GmTIP1;8* under stress conditions, these genes were transformed into yeast cells. The overexpressing yeast cells were grown in media either containing 5 M NaCl (salt stress) or 30% PEG (dehydration stress). We observed that the yeast cells overexpressing each construct showed better growth compared to the control yeast cells (transformed with an empty vector under salt and drought conditions, respectively), however yeast cells harboring *GmTIP2;1* overexpressing constructs performed best under drought stress conditions (Fig. 6a). We also measured the growth rate of overexpressing yeast cells by performing growth curve analysis, which indicated that the yeast cells overexpressing *GmTIP2*;*1*, *GmTIP1*;*7* and *GmTIP1*;*8* had faster growth compared to control cells under salt and drought treatments, respectively (Fig. 6b,c). Taken together, these results confirmed that overexpression of GmTIP2;1, GmTIP1;7 and GmTIP1;8 could improve salt and dehydration tolerance.

### *In-planta* overexpression of *GmTIP2;1*, *GmTIP1;7* and *Gm TIP1;8* improved salt tolerance

We further validated these genes for their ability to provide tolerance to stress conditions by overexpressing these *in-planta* under constitutive promoter. The transgenic plants were treated with 200 mM NaCl solution for seven days and observed for phenotypic changes. The *GmTIP2*;*1*, *GmTIP1*;*7*, *GmTIP1*;*8* overexpressing transgenics did not show any sign of wilting in 200 mM NaCl containing hydroponic media, however, the wilting was observed in empty vector control transgenic plants (Fig. 6d). We also quantified the expression of these AQPs in transgenic plants, which revealed that *GmTIP2*;*1*, *GmTIP1*;*7* and *GmTIP1*;*8* were up regulated under stress conditions (Fig. 6e). This suggests that *GmTIP2*;*1*, *GmTIP1*;*7* and *GmTIP1*;*8* are involved in imparting stress tolerance to soybean plants in hydroponic media.

## Discussion

Abiotic stress is a major environmental factor affecting crop productivity and yield. Amongst many forms of abiotic stresses, soil salinity and dehydration stress significantly affect plant growth and yield. AQPs are responsible for water transport across membranes, thus playing important regulatory roles in the salt and osmotic stress responses in plants. It has been known that *Glycine soja*, a wild relative of cultivated soybean is relatively stress tolerant^24^, thus we aimed to identify *AQP* genes from this species and their potential involvement and functions towards stress tolerance. In the present study, we identified 62 *GsAQP* genes using HMM model, which is in agreement with number of *GmAQPs* suggesting that aquaporin genes did not go through much of the duplication during domestication. These proteins ranged between 160–380 amino acids and contained 4–7 transmembrane regions (TMR) and 1–3 NPA motifs. Classically AQPs have six TMRs, with small hydrophilic loops connecting these regions and two highly conserved NPA motifs in extramembrane peptide loops thought to be involved in channel selectivity^25^, however higher plants have shown several diversification in their structure^1^, which could be due to their specific requirements for relatively quick fine tuned adaptation in a rapidly changing environment, especially dehydration or flooding. As *Glycine soja* is known to be stress tolerant, the structural variance in TMR and NPA motifs could be involved in forming efficient water transport channel leading to cope up stress better.

There have been reports of varying *AQP* gene expression patterns in specific tissues and cell types of many plant species as well as in response to environmental factors, suggesting the diverse functions of water channels^26^. Thus, we analyzed spatial expression of *GmAQP* genes using RNA-seq based transcriptome data and found that their expressions varied among different tissues. Clustering analysis revealed two clusters, one having genes up regulated in most tissues including roots and second having genes exclusively up regulated in roots. The genes present in these clusters most likely explain two scenarios, first: only root specific genes are probably involved in capturing water from soil where as the genes present in second cluster are involved in water and solute transport through out the plant body. The cluster having genes expressed in most of the tissues include four PIPs and two TIPs, which is in accordance with earlier report suggesting PIPs are generally localized in organs and tissues characterized by large fluxes of water^27^. On the other hand the root specific cluster contain genes from NIPs (2), TIPs (3) and PIPs (2), out of which NIPs have not yet been reported in soil related stress conditions and PIPs expression is relatively low compared to TIPs. Taken together, we found four TIPs, which are differentially up regulated in roots as well as in other tissues, namely *GmTIP2;1*, *GmTIP1;7*, *GmTIP1;8* and *GmTIP4;1*, however, upon further analysis it was observed that *GmTIP4;1* has least expression amongst all (Fig. 4c). After spatial expression analysis, we decided to examine the expression of *GmAQPs* under both salt and dehydration stress conditions and both of which are mediated by water that usually gets transported through AQPs.

The transcriptome analysis data revealed variable expression pattern for most of the *AQP* genes in response to salt and dehydration stress, however we found that *GmTIP2*;*1* and *GmTIP1*;*7* are consistently up regulated under both stress conditions and *GmTIP1*;*8* was up regulated in dehydration but down regulated in salt stress. This suggested that these three genes are probably the major *AQPs* involved in salt and dehydration stress. To further examine and validate these genes in both species, we analyzed their respective expression qualitatively in roots and leaves under salinity and dehydration stress. We observed that most of the PIPs and TIPs are relatively up regulated in *Glycine soja* tissues, suggesting probably better water transport mechanism leading to stress tolerance. Notably, we also observed that expression of *GmTIP2*;*1*, *GmTIP1*;*7* and *GmTIP1*;*8* is also consistent with transcriptome data. Taken together, the expression analysis confirmed the differential expression of AQPs at tissue level in *Glycine max* as well as between both the varieties, thus we decided to analyze their functional role.

To address this, we performed protein-protein interaction studies and found that GmTIP2;1 interacts with itself as well as with GmTIP1;7 and/or GmTIP1;8, possibly suggesting an intricate and complex level of regulation. Aquaporins are known to be assembled as tetramers; the four subunits are arranged in parallel, forming a fifth pore in the center of the tetramer to facilitate water flow^2^. Additionally, it has been reported that the function of AQPs is controlled by physiological signals as well as the interactions between different AQP monomers or interaction with other proteins^28^. Further, the role of hetero-molecular AQP interactions has also been described^9, 29^ and likewise, heteroligomerization between PIP1 and PIP2 has been shown to modify the characteristics of water channels^30, 31^. In case of GmTIPs, the sequences of GmTIP1;7 and GmTIP1;8 were found to be very similar to that of GmTIP2;1, except for differences of few amino acids at the ends of the C-termini. Taken together, we can conclude that GmTIP2;1 interacts with itself as well as GmTIP1;7 and GmTIP1;8, which possibly leads to different physiological functions under stress conditions. Moreover at cellular level, we could validate that overexpression of *GmTIP2;1* and *GmTIP1;7* or *GmTIP1;8* lead to improved salt and drought tolerance in yeast, which further suggested that these genes could be important players in salt and dehydration tolerance in plants as well. We validated this using transgenic plants overexpressing respective genes, which showed salt tolerance, suggesting these genes are indeed involved in stress response pathway and might be responsible for protection of plants under adverse conditions. This result is in contrast to the previous report suggesting that overexpression of *Glycine soja* *GsTIP2;1* in Arabidopsis reduces tolerance to salt and dehydration stresses^20^. This probably suggests diverse regulation of TIPs within the species, such as *GsTIP2;1* overexpression results are opposite to another study, which demonstrated that a *Panax ginseng* tonoplast aquaporin gene (*PgTIP1*), promoted plant growth under favorable conditions, enhanced salt stress tolerance and diminished chilling tolerance in transgenic Arabidopsis^14^.

Taken together, in the present study, we have identified *AQP* genes from wild soybean, as well as performed transcriptome based analysis to identify differentially expressed *GmAQPs* at tissue level and under stress conditions;  namely salt and drought. We further identified three key genes involved in salt stress and characterized their functions. Moreover, we could also show that overexpression of these genes could enhance salinity and drought tolerance *in-planta*. In the era of continuously changing environment, we need to find novel candidate genes that can be employed to develop strategies to enhance the tolerance of crops from abiotic stresses such as salinity and water limitation conditions. Overall, a more comprehensive understanding will emerge as and when multiple aquaporin transport functions and integration of the different stress signals will be established at plant level.

## Methods

### Plant materials, bacteria and reagents

The soybean variety Williams 82 (*Glycine max*) and wild soybean (*Glycine soja*, *STGoGS*) were obtained from coastal saline soil in Jiangsu province, China. The *Escherichia coli* DH5 alpha was preserved at our laboratory. A pGEM-T Easy vector cloning kit, PrimeSTAR Taq DNA polymerase, DNA ladder, TRIzol reagent and T4 DNA ligase were purchased from Fermentas. The reverse transcription kit was obtained from Shanghai Generay Bioengineering Co., Ltd. The DNA purification kit was acquired from Promega and additional biological reagents were procured from Shanghai Hao Jia Technology Development Co., Ltd.

### Sequence identification and bioinformatics analysis

The GmAQP sequences described in our previous work^13^ were used as a template for identification of GsAQPs. We performed multiple sequence alignment of all GmAQPs and used this as an input to generate HMM profile using HMMER software. We used that profile as default parameters in the HMM search program of the HMMER package against *Glycine soja* proteome (Uniprot id.UP000053555). All the significant hits with positive scores were selected for classification and were then examined individually for accessory domains. All the obtained hits were individually confirmed by BLAST against GmAQPs using BLASTP. Sequence analysis, motif detection and transmembrane region analysis was carried out using Geneious Version 7 software with default parameters.

### Expression Profiling Using RNA-seq Datasets

The RNA-seq data generated previously for fourteen different tissues including flower, leaves, nodules, pod, root, seed etc. as well as of salinity and drought treated plant materials were downloaded from soybase (https://soybase.org/soyseq/) and used to analyze expression patterns of *GmAQPs*. The expression values for AQPs in response to stresses were obtained through previously published work of Belamakar *et al*. (2014) (GEO accession no. GSE57252). We considered a gene was expressed if the RPKM value was greater than or equal to two in an expression atlas. The RPKM normalized read count data of expressed genes was log2-transformed and displayed in the form of heatmaps and clustered using R software.

### Total RNA extraction and cDNA synthesis

Root and leaf samples from cultivated and wild soybean seedlings at the three-leaf stage were treated with 200 mM NaCl for 0, 0.5, 1, 3, 6, 12 and 24 h and samples were harvested and grounded in liquid nitrogen. Total RNA was extracted using Plant RNA extraction kit (Promega, Beijing, China) according to the manufacturer’s instructions. Single-stranded cDNA was synthesized from 1 µg of total RNA with Oligo (dT)18 primers in a total volume of 20 µl using a Takara RT-PCR system according to the suppliers instructions.

### Semi-quantitative RT-PCR

Gene expression was analyzed by performing semi-quantitative RT-PCR, using the soybean housekeeping gene *GmActin* (*Glycine max* (*actin-1-like*), GenBank Accession No: XM_003552652) as a control, and the primers used to target the *AQPs* are listed in Supplementary Table S2. PCR was carried out with 1 μl cDNA sample in 25 μl of PCR mix, containing 2.5 μL 10 × PCR buffer, 0.5 μL of 10 mM dNTPs, 1.5 μL of 25 mMgCl_2_, 1 μL of each primer, and 0.2 μL of 5 U mL^−1^ Taq DNA polymerase. The PCR conditions were as follows: 94 °C for 3 min, followed by 26~29 cycles of 45 seconds denaturation at 94 °C, 45 seconds primer annealing at 55 °C and 30 s extension at 72 °C, with a final extension step at 72 °C for 10 min.

### Yeast two-hybrid assay

The full-length ORFs of AQPs were cloned using primers having *Nco1* and *EcoRI* restriction sites (the primers used are listed in Supplementary Table S3) and ligated into pGADT7 and pGBKT7 vectors, respectively. The yeast two-hybrid assay was performed according to the Matchmaker^TM^ Gold Yeast Two-Hybrid System protocol (Clontech).

### Yeast transformation and treatments

The coding sequences (CDS) of *GmTIP2*;*1*, *GmTIP1*;*7*, and *GmTIP1*;*8*, including the *Hind III* and *Xbal* restriction sites for the forward and reverse primers (Supplementary Table S3), respectively, were inserted into a pYES2 vector digested using these two enzymes. The resulting clones were introduced into *S*. *cerevisiae* INVSc1 strain cells using the lithium acetate method, and an empty pYES2 vector was used as a control. *S*. *cerevisiae* INVSc1 strain cells were transformed with an empty pYES2 vector only, pYES2-*GmTIP2;1*, pYES2-*GmTIP1;7*, pYES2-*GmTIP1;8*, and combinations of these three vectors after induction with galactose. These were then spotted onto SC-Ura medium at 1-, 10-, 100-, and 1000-fold dilutions. The expression effect of these genes in yeast cells in response to salinity and drought was analyzed after treatment with 5 M NaCl and 30% PEG 6000 for 40 h respectively.

### Bimolecular fluorescence complementation (BiFC)

The full-length CDS of *GmTIP2*;*1*, *GmTIP1*;*7* and *GmTIP1*;*8* were amplified using primers in Supplementary Table S3, and were then cloned into the pSPYNE-35S vector, which contains the N-terminus of YFP. The full-length cDNA of *GmTIP2;1* was amplified and cloned into pSPYCE-35S vector, which contains the C-terminus of YFP. The constructs were introduced into the *Agrobacterium tumefaciens* strain GV3101 by electroporation. Agrobacterium cells carrying pSPYNE-35S and pSPYCE-35S were inoculated, harvested and resuspended in infiltration media (10 mM MgCl2, 10 mM MES, 200 µM acetosyringone) and adjusted to OD600 at 1.0. The Agrobacterium strains containing pSPYNE-35S and pSPYCE-35S vectors were mixed at a ratio of 1:1. The cotyledons of 7 days old *Nocotianna benthimiana* seedlings were injected by the Agrobacterium suspension and then incubated at 25 °C. The YFP fluorescence was detected 48 h after transfection using a ZEISS LSM 710 confocal microscope (Zeiss Microsystems).

### Yeast growth curve measurement assay

Growth curves were measured by microcultivation experiments in triplicate at 30 °C using the Bioscreen C system (Labsystems Oy, Helsinki, Finland). Overnight grown yeast cultures with OD value 0.8 were induced by galactose and inoculated in 3 ml YPDA with addition of NaCl (0.5 M) or PEG (10%). The optical density was measured every 2 h for 48 h. Raw data in triplicate were used to get average values for plotting in Excel software.

### Overexpression of GmAQPs

The overexpression analysis was performed using hairy root system to characterize the function of salt response. These genes were cloned using pCXSN vector^32^ containing double tobacco mosaic virus promoter 35S and transformed using Agrobacterium method. The successful transformation of seedlings resulted in developing hairy roots and the plants having such roots were used further for overexpression analysis. The day before transformation, *Agrobacterium* carrying gene of interest was grown overnight in antibiotic containing media. The *Agrobacterium* was injected at juncture of two cotyledons and the inject point was covered with soil. The cultivation of transgenic plants and treatment method were carried out according to the Ali *et al*.^33^. The development of hairy roots took around 10–15 days after injection and after that the true roots of plants were cut-off below the inject point from where hairy roots developed. Only one hairy root was kept and rest were removed by cutting. The seedlings were allowed to grow in glass-tubes containing 1/2 strength Hoagland solution in growth chamber set at day/night temperatures of 28/25 ± 2 °C and photoperiod of 12 h. After one week the control and transformed mosaic soybean seedlings were treated with 200 mM NaCl and evaluated on 7^th^ day of stress. The candidate gene expression was analyzed by real time quantitative PCR using SYBR Green Supermix (Takara) and the reaction was carried out in Roche 2.0 Real-Time PCR Detection System. The primers are listed in the Supplementary Table.

## Electronic supplementary material

Supplementary material
