# Author Correction: Genome-wide identification of Major Intrinsic Proteins in *Glycine soja* and characterization of GmTIP2;1 function under salt and water stress

**DOI:** 10.1038/s41598-020-59132-x

**Published:** 2020-02-06

**Authors:** Da-yong Zhang, Manoj Kumar, Ling Xu, Qun Wan, Yi-hong Huang, Zhao-Long Xu, Xiao-Lan He, Jin-Biao Ma, Girdhar K. Pandey, Hong-Bo Shao

**Affiliations:** 1 Salt-soil Agricultural Center, Institute of Agricultural Resources and Environment, Jiangsu Academy of Agricultural Sciences, Zhongling Street No.50, Nanjing, 210014 China; 2 Department of Plant Molecular Biology, University of Delhi South Campus, Benito Juarez Road, Dhaula Kuan, New Delhi 110021 India; 3 Key Laboratory of Biogeography and Bioresources in Arid Land, Xinjiang Institute of Ecology and Geography, Chinese Academy of Sciences Urumqi, Urumqi, China; 4 JLCBE, Yancheng Teachers University, Xiwang Avenue 1, Yancheng, 224002 China; 5 School of Biotechnology, Jawaharlal Nehru University, New Delhi, 110067 India

Correction to: *Scientific Reports* 10.1038/s41598-017-04253-z, published online 23 June 2017

This Article contains errors in Figure 4a, where the GmPIP2;11 gel panels of *Glycine max* root, *Glycine max* leaf and *Glycine soja* leaf were mistakenly duplicated. These gel panels have now been corrected. The correct Figure 4 appears below as Figure 1.

**Figure 1:**
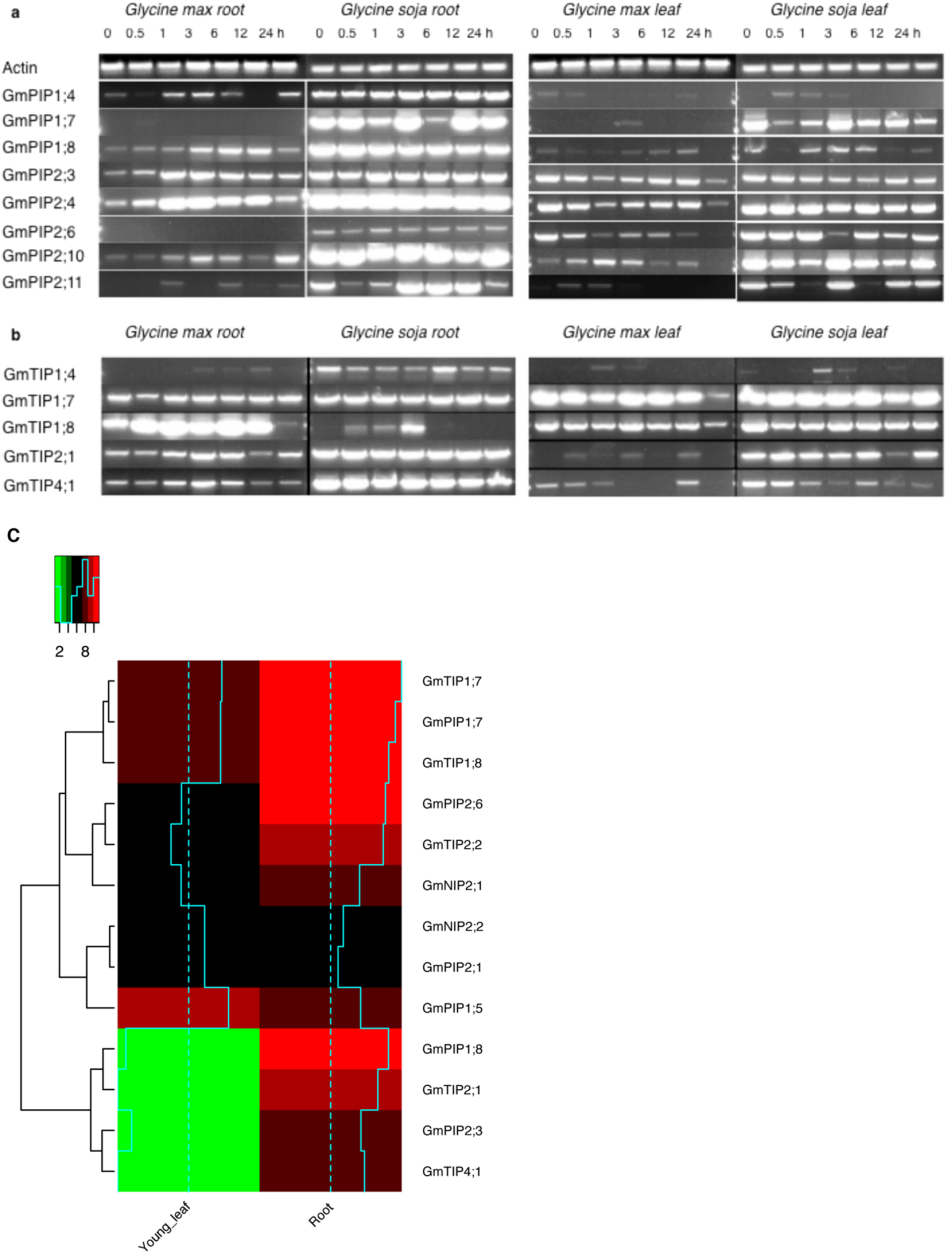
.

