# Supplementary material for: Genome-wide identification of Major Intrinsic Proteins in Glycine soja and characterization of GmTIP2;1 function under salt and water stress
Source: Sci Rep. 2017 Jun 23;7:4106. doi: 10.1038/s41598-017-04253-z (PMC5482899; doi:10.1038/s41598-017-04253-z)
Supplement: Supplementary file 1 — Supplementary material [file 41598_2017_4253_MOESM1_ESM.pdf]

Supplementary materials

# Genome-wide identification of Major Intrinsic Proteins in *Glycine soja* and characterization of GmTIP2;1 function under salt and water stress

Da-yong Zhang [1,#], Manoj Kumar[2,5,#], Ling Xu [1], Qun Wan[1], Yi-hong Huang [1], Zhao-Long Xu [1], Xiao-Lan He [1], Jin-Biao Ma [3], Girdhar K. Pandey[2,*], Hong-Bo Shao [1,4,*]

1. Salt-soil Agricultural Center, Institute of Agricultural Resources and Environment, Jiangsu Academy of Agricultural Sciences, Zhongling Street No.50, Nanjing 210014, China
2. Department of Plant Molecular Biology, University of Delhi South Campus, Benito Juarez Road, Dhaula Kuan, New Delhi-110021, India.
3. Key Laboratory of Biogeography and Bioresources in Arid Land, Xinjiang Institute of Ecology and Geography, Chinese Academy of Sciences Urumqi, China.
4. JLCBE, Yancheng Teachers University, Xiwang Avenue 1, Yancheng 224002, China.
5. School of Biotechnology, Jawaharlal Nehru University, New Delhi, India.

[#] These authors equally contributed

*Author for correspondence: Email: gkpandey@south.du.ac.in; shaohongbochu@126.com

Tel.: +91-9811721436; +86 25 84391105; Fax: +86 25 84390297

Supplementary Table S1

Motifs and Domains in GsAQPs

| Gene Name | Motif/Domain Name | Start Position | End Position | Length |
|---|---|---|---|---|
| GsXIP1;1 | NPA | 183 | 185 | 3 |
| GsNIP7;1 | NPA | 57 | 59 | 3 |
| GsNIP7;1 | NPA | 168 | 170 | 3 |
| GsNIP7;2 | NPA | 47 | 49 | 3 |
| GsNIP7;2 | NPA | 158 | 160 | 3 |
| GsNIP2;2 | NPA | 110 | 112 | 3 |
| GsNIP2;2 | NPA | 221 | 223 | 3 |
| GsNIP2;1 | NPA | 108 | 110 | 3 |
| GsNIP2;1 | NPA | 220 | 222 | 3 |
| GsNIP6;2 | NPA | 131 | 133 | 3 |
| GsNIP6;1 | NPA | 136 | 138 | 3 |
| GsNIP1;4 | NPA | 100 | 102 | 3 |
| GsNIP1;4 | NPA | 212 | 214 | 3 |
| GsNIP1;3 | NPA | 100 | 102 | 3 |
| GsNIP1;3 | NPA | 212 | 214 | 3 |
| GsNIP1;1 | NPA | 97 | 99 | 3 |
| GsNIP1;1 | NPA | 209 | 211 | 3 |
| GsNIP1;2 | NPA | 97 | 99 | 3 |
| GsNIP1;2 | NPA | 209 | 211 | 3 |
| GsNIP1;5 | NPA | 97 | 99 | 3 |
| GsNIP1;5 | NPA | 209 | 211 | 3 |
| GsNIP4;1 | NPA | 102 | 104 | 3 |
| GsNIP4;1 | NPA | 214 | 216 | 3 |
| GsPIP1;3 | NPA | 116 | 118 | 3 |
| GsPIP1;3 | NPA | 237 | 239 | 3 |
| GsPIP1;2 | NPA | 113 | 115 | 3 |
| GsPIP1;2 | NPA | 234 | 236 | 3 |
| GsPIP1;1 | NPA | 113 | 115 | 3 |
| GsPIP1;1 | NPA | 234 | 236 | 3 |
| GsPIP1;5 | NPA | 113 | 115 | 3 |
| GsPIP1;5 | NPA | 235 | 237 | 3 |
| GsPIP1;4 | NPA | 113 | 115 | 3 |
| GsPIP1;4 | NPA | 235 | 237 | 3 |
| GsPIP1;6 | NPA | 114 | 116 | 3 |
| GsPIP1;6 | NPA | 236 | 238 | 3 |
| GsPIP1;7 | NPA | 117 | 119 | 3 |
| GsPIP1;7 | NPA | 239 | 241 | 3 |
| GsPIP1;8 | NPA | 117 | 119 | 3 |
| GsPIP1;8 | NPA | 239 | 241 | 3 |
| GsPIP2;8 | NPA | 14 | 16 | 3 |

| | | | | |
|---|---|---|---|---|
| GsPIP2;8 | NPA | 135 | 137 | 3 |
| GsPIP2;1 | NPA | 95 | 97 | 3 |
| GsPIP2;1 | NPA | 216 | 218 | 3 |
| GsPIP2;11 | NPA | 59 | 61 | 3 |
| GsPIP2;11 | NPA | 180 | 182 | 3 |
| GsPIP2;9 | NPA | 105 | 107 | 3 |
| GsPIP2;9 | NPA | 226 | 228 | 3 |
| GsPIP2;10 | NPA | 105 | 107 | 3 |
| GsPIP2;10 | NPA | 226 | 228 | 3 |
| GsPIP2;10 | NPA | 282 | 284 | 3 |
| GsPIP2;14 | NPA | 104 | 106 | 3 |
| GsPIP2;14 | NPA | 225 | 227 | 3 |
| GsPIP2;13 | NPA | 107 | 109 | 3 |
| GsPIP2;13 | NPA | 228 | 230 | 3 |
| GsPIP2;3 | NPA | 108 | 110 | 3 |
| GsPIP2;3 | NPA | 229 | 231 | 3 |
| GsPIP2;4 | NPA | 108 | 110 | 3 |
| GsPIP2;4 | NPA | 229 | 231 | 3 |
| GsPIP2;6 | NPA | 109 | 111 | 3 |
| GsPIP2;6 | NPA | 230 | 232 | 3 |
| GsPIP2;5 | NPA | 109 | 111 | 3 |
| GsPIP2;5 | NPA | 230 | 232 | 3 |
| GsTIP5;1 | NPA | 44 | 46 | 3 |
| GsTIP5;1 | NPA | 186 | 188 | 3 |
| GsTIP3;1 | NPA | 85 | 87 | 3 |
| GsTIP3;1 | NPA | 199 | 201 | 3 |
| GsTIP3;2 | NPA | 85 | 87 | 3 |
| GsTIP3;2 | NPA | 199 | 201 | 3 |
| GsTIP3;3 | NPA | 85 | 87 | 3 |
| GsTIP3;3 | NPA | 199 | 201 | 3 |
| GsTIP3;4 | NPA | 85 | 87 | 3 |
| GsTIP3;4 | NPA | 199 | 201 | 3 |
| GsTIP4;2 | NPA | 79 | 81 | 3 |
| GsTIP4;2 | NPA | 193 | 195 | 3 |
| GsTIP4;1 | NPA | 79 | 81 | 3 |
| GsTIP4;1 | NPA | 193 | 195 | 3 |
| GsTIP1;4 | NPA | 85 | 87 | 3 |
| GsTIP1;4 | NPA | 199 | 201 | 3 |
| GsTIP1;5 | NPA | 85 | 87 | 3 |
| GsTIP1;5 | NPA | 199 | 201 | 3 |
| GsTIP1;6 | NPA | 85 | 87 | 3 |
| GsTIP1;6 | NPA | 199 | 201 | 3 |
| GsTIP1;7 | NPA | 44 | 46 | 3 |
| GsTIP1;3 | NPA | 16 | 18 | 3 |
| GsTIP1;3 | NPA | 85 | 87 | 3 |
| GsTIP1;3 | NPA | 199 | 201 | 3 |

| | | | | |
|---|---|---|---|---|
| GsTIP1;1 | NPA | 85 | 87 | 3 |
| GsTIP1;1 | NPA | 199 | 201 | 3 |
| GsTIP1;2 | NPA | 85 | 87 | 3 |
| GsTIP1;2 | NPA | 199 | 201 | 3 |
| GsTIP2;1 | NPA | 83 | 85 | 3 |
| GsTIP2;1 | NPA | 196 | 198 | 3 |
| GsTIP2;2 | NPA | 83 | 85 | 3 |
| GsTIP2;2 | NPA | 196 | 198 | 3 |
| GsTIP2;4 | NPA | 83 | 85 | 3 |
| GsTIP2;4 | NPA | 197 | 199 | 3 |
| GsTIP2;3 | NPA | 83 | 85 | 3 |
| GsTIP2;3 | NPA | 197 | 199 | 3 |
| GsTIP2;7 | NPA | 83 | 85 | 3 |
| GsTIP2;7 | NPA | 196 | 198 | 3 |
| GsTIP2;6 | NPA | 83 | 85 | 3 |
| GsTIP2;6 | NPA | 196 | 198 | 3 |
| GsTIP2;5 | NPA | 83 | 85 | 3 |
| GsTIP2;5 | NPA | 196 | 198 | 3 |
| GsSIP2;1 | NPA | 179 | 181 | 3 |
| GsSIP1;6 | NPA | 188 | 190 | 3 |
| GsSIP1;5 | NPA | 188 | 190 | 3 |
| GsSIP1;4 | NPA | 193 | 195 | 3 |
| GsSIP1;3 | NPA | 193 | 195 | 3 |
| GsSIP1;2 | NPA | 193 | 195 | 3 |
| GsSIP1;1 | NPA | 247 | 249 | 3 |
| GsXIP1;2 | Transmembrane | 120 | 140 | 21 |
| GsXIP1;2 | Transmembrane | 79 | 99 | 21 |
| GsXIP1;2 | Transmembrane | 49 | 70 | 22 |
| GsXIP1;2 | Transmembrane | 174 | 194 | 21 |
| GsXIP1;2 | Transmembrane | 207 | 227 | 21 |
| GsXIP1;2 | Transmembrane | 257 | 277 | 21 |
| GsXIP1;1 | Transmembrane | 62 | 82 | 21 |
| GsXIP1;1 | Transmembrane | 91 | 111 | 21 |
| GsXIP1;1 | Transmembrane | 21 | 41 | 21 |
| GsXIP1;1 | Transmembrane | 120 | 140 | 21 |
| GsXIP1;1 | Transmembrane | 153 | 173 | 21 |
| GsXIP1;1 | Transmembrane | 178 | 198 | 21 |
| GsXIP1;1 | Transmembrane | 201 | 221 | 21 |
| GsNIP7;1 | Transmembrane | 75 | 95 | 21 |
| GsNIP7;1 | Transmembrane | 186 | 206 | 21 |
| GsNIP7;1 | Transmembrane | 145 | 165 | 21 |
| GsNIP7;1 | Transmembrane | 34 | 54 | 21 |
| GsNIP7;1 | Transmembrane | 3 | 23 | 21 |
| GsNIP7;1 | Transmembrane | 112 | 132 | 21 |
| GsNIP7;2 | Transmembrane | 66 | 86 | 21 |
| GsNIP7;2 | Transmembrane | 176 | 196 | 21 |

| | | | | |
|---|---|---|---|---|
| GsNIP7;2 | Transmembrane | 135 | 155 | 21 |
| GsNIP7;2 | Transmembrane | 17 | 37 | 21 |
| GsNIP7;2 | Transmembrane | 43 | 63 | 21 |
| GsNIP7;2 | Transmembrane | 102 | 122 | 21 |
| GsNIP2;2 | Transmembrane | 125 | 146 | 22 |
| GsNIP2;2 | Transmembrane | 238 | 258 | 21 |
| GsNIP2;2 | Transmembrane | 196 | 217 | 22 |
| GsNIP2;2 | Transmembrane | 84 | 104 | 21 |
| GsNIP2;2 | Transmembrane | 53 | 73 | 21 |
| GsNIP2;2 | Transmembrane | 167 | 187 | 21 |
| GsNIP2;1 | Transmembrane | 124 | 145 | 22 |
| GsNIP2;1 | Transmembrane | 237 | 257 | 21 |
| GsNIP2;1 | Transmembrane | 195 | 216 | 22 |
| GsNIP2;1 | Transmembrane | 83 | 103 | 21 |
| GsNIP2;1 | Transmembrane | 51 | 71 | 21 |
| GsNIP2;1 | Transmembrane | 166 | 186 | 21 |
| GsNIP5;1 | Transmembrane | 108 | 129 | 22 |
| GsNIP5;1 | Transmembrane | 218 | 238 | 21 |
| GsNIP5;1 | Transmembrane | 176 | 197 | 22 |
| GsNIP5;1 | Transmembrane | 67 | 87 | 21 |
| GsNIP5;1 | Transmembrane | 33 | 53 | 21 |
| GsNIP5;1 | Transmembrane | 143 | 163 | 21 |
| GsNIP6;2 | Transmembrane | 152 | 172 | 21 |
| GsNIP6;2 | Transmembrane | 259 | 280 | 22 |
| GsNIP6;2 | Transmembrane | 218 | 238 | 21 |
| GsNIP6;2 | Transmembrane | 103 | 123 | 21 |
| GsNIP6;2 | Transmembrane | 74 | 94 | 21 |
| GsNIP6;2 | Transmembrane | 189 | 209 | 21 |
| GsNIP6;1 | Transmembrane | 282 | 302 | 21 |
| GsNIP6;1 | Transmembrane | 108 | 128 | 21 |
| GsNIP6;1 | Transmembrane | 235 | 255 | 21 |
| GsNIP6;1 | Transmembrane | 79 | 99 | 21 |
| GsNIP6;1 | Transmembrane | 194 | 214 | 21 |
| GsNIP1;4 | Transmembrane | 118 | 138 | 21 |
| GsNIP1;4 | Transmembrane | 230 | 250 | 21 |
| GsNIP1;4 | Transmembrane | 188 | 209 | 22 |
| GsNIP1;4 | Transmembrane | 77 | 97 | 21 |
| GsNIP1;4 | Transmembrane | 43 | 63 | 21 |
| GsNIP1;4 | Transmembrane | 159 | 179 | 21 |
| GsNIP1;3 | Transmembrane | 118 | 138 | 21 |
| GsNIP1;3 | Transmembrane | 230 | 250 | 21 |
| GsNIP1;3 | Transmembrane | 188 | 209 | 22 |
| GsNIP1;3 | Transmembrane | 77 | 97 | 21 |
| GsNIP1;3 | Transmembrane | 43 | 63 | 21 |
| GsNIP1;3 | Transmembrane | 159 | 179 | 21 |
| GsNIP1;1 | Transmembrane | 115 | 135 | 21 |

| | | | | |
|---|---|---|---|---|
| GsNIP1;1 | Transmembrane | 33 | 53 | 21 |
| GsNIP1;1 | Transmembrane | 225 | 245 | 21 |
| GsNIP1;1 | Transmembrane | 184 | 204 | 21 |
| GsNIP1;1 | Transmembrane | 74 | 94 | 21 |
| GsNIP1;1 | Transmembrane | 156 | 175 | 20 |
| GsNIP1;2 | Transmembrane | 115 | 135 | 21 |
| GsNIP1;2 | Transmembrane | 225 | 245 | 21 |
| GsNIP1;2 | Transmembrane | 184 | 204 | 21 |
| GsNIP1;2 | Transmembrane | 74 | 94 | 21 |
| GsNIP1;2 | Transmembrane | 40 | 60 | 21 |
| GsNIP1;2 | Transmembrane | 156 | 175 | 20 |
| GsNIP1;5 | Transmembrane | 112 | 133 | 22 |
| GsNIP1;5 | Transmembrane | 225 | 245 | 21 |
| GsNIP1;5 | Transmembrane | 184 | 204 | 21 |
| GsNIP1;5 | Transmembrane | 72 | 91 | 20 |
| GsNIP1;5 | Transmembrane | 154 | 175 | 22 |
| GsNIP1;5 | Transmembrane | 40 | 60 | 21 |
| GsNIP4;1 | Transmembrane | 120 | 140 | 21 |
| GsNIP4;1 | Transmembrane | 232 | 252 | 21 |
| GsNIP4;1 | Transmembrane | 190 | 211 | 22 |
| GsNIP4;1 | Transmembrane | 73 | 93 | 21 |
| GsNIP4;1 | Transmembrane | 50 | 70 | 21 |
| GsNIP4;1 | Transmembrane | 161 | 181 | 21 |
| GsPIP1;3 | Transmembrane | 135 | 155 | 21 |
| GsPIP1;3 | Transmembrane | 259 | 279 | 21 |
| GsPIP1;3 | Transmembrane | 99 | 126 | 28 |
| GsPIP1;3 | Transmembrane | 209 | 229 | 21 |
| GsPIP1;3 | Transmembrane | 58 | 78 | 21 |
| GsPIP1;3 | Transmembrane | 176 | 196 | 21 |
| GsPIP1;2 | Transmembrane | 131 | 152 | 22 |
| GsPIP1;2 | Transmembrane | 256 | 276 | 21 |
| GsPIP1;2 | Transmembrane | 90 | 110 | 21 |
| GsPIP1;2 | Transmembrane | 206 | 226 | 21 |
| GsPIP1;2 | Transmembrane | 54 | 74 | 21 |
| GsPIP1;2 | Transmembrane | 173 | 193 | 21 |
| GsPIP1;1 | Transmembrane | 131 | 152 | 22 |
| GsPIP1;1 | Transmembrane | 255 | 275 | 21 |
| GsPIP1;1 | Transmembrane | 90 | 110 | 21 |
| GsPIP1;1 | Transmembrane | 206 | 226 | 21 |
| GsPIP1;1 | Transmembrane | 54 | 74 | 21 |
| GsPIP1;1 | Transmembrane | 173 | 193 | 21 |
| GsPIP1;5 | Transmembrane | 131 | 152 | 22 |
| GsPIP1;5 | Transmembrane | 257 | 277 | 21 |
| GsPIP1;5 | Transmembrane | 206 | 226 | 21 |
| GsPIP1;5 | Transmembrane | 90 | 110 | 21 |
| GsPIP1;5 | Transmembrane | 54 | 74 | 21 |

| | | | | |
|---|---|---|---|---|
| GsPIP1;5 | Transmembrane | 173 | 193 | 21 |
| GsPIP1;4 | Transmembrane | 131 | 152 | 22 |
| GsPIP1;4 | Transmembrane | 257 | 277 | 21 |
| GsPIP1;4 | Transmembrane | 206 | 226 | 21 |
| GsPIP1;4 | Transmembrane | 90 | 110 | 21 |
| GsPIP1;4 | Transmembrane | 54 | 74 | 21 |
| GsPIP1;4 | Transmembrane | 173 | 193 | 21 |
| GsPIP1;6 | Transmembrane | 132 | 153 | 22 |
| GsPIP1;6 | Transmembrane | 258 | 278 | 21 |
| GsPIP1;6 | Transmembrane | 207 | 227 | 21 |
| GsPIP1;6 | Transmembrane | 91 | 111 | 21 |
| GsPIP1;6 | Transmembrane | 55 | 75 | 21 |
| GsPIP1;6 | Transmembrane | 174 | 194 | 21 |
| GsPIP1;7 | Transmembrane | 135 | 156 | 22 |
| GsPIP1;7 | Transmembrane | 261 | 281 | 21 |
| GsPIP1;7 | Transmembrane | 94 | 114 | 21 |
| GsPIP1;7 | Transmembrane | 211 | 231 | 21 |
| GsPIP1;7 | Transmembrane | 61 | 81 | 21 |
| GsPIP1;8 | Transmembrane | 135 | 156 | 22 |
| GsPIP1;8 | Transmembrane | 261 | 281 | 21 |
| GsPIP1;8 | Transmembrane | 94 | 114 | 21 |
| GsPIP1;8 | Transmembrane | 211 | 231 | 21 |
| GsPIP1;8 | Transmembrane | 61 | 81 | 21 |
| GsPIP2;8 | Transmembrane | 33 | 53 | 21 |
| GsPIP2;8 | Transmembrane | 157 | 177 | 21 |
| GsPIP2;8 | Transmembrane | 107 | 127 | 21 |
| GsPIP2;8 | Transmembrane | 74 | 94 | 21 |
| GsPIP2;1 | Transmembrane | 108 | 135 | 28 |
| GsPIP2;1 | Transmembrane | 189 | 209 | 21 |
| GsPIP2;1 | Transmembrane | 67 | 87 | 21 |
| GsPIP2;1 | Transmembrane | 156 | 176 | 21 |
| GsPIP2;1 | Transmembrane | 35 | 55 | 21 |
| GsPIP2;11 | Transmembrane | 78 | 98 | 21 |
| GsPIP2;11 | Transmembrane | 202 | 222 | 21 |
| GsPIP2;11 | Transmembrane | 26 | 46 | 21 |
| GsPIP2;11 | Transmembrane | 152 | 172 | 21 |
| GsPIP2;11 | Transmembrane | 49 | 69 | 21 |
| GsPIP2;11 | Transmembrane | 119 | 139 | 21 |
| GsPIP2;9 | Transmembrane | 124 | 144 | 21 |
| GsPIP2;9 | Transmembrane | 248 | 268 | 21 |
| GsPIP2;9 | Transmembrane | 72 | 92 | 21 |
| GsPIP2;9 | Transmembrane | 198 | 218 | 21 |
| GsPIP2;9 | Transmembrane | 95 | 115 | 21 |
| GsPIP2;9 | Transmembrane | 39 | 59 | 21 |
| GsPIP2;9 | Transmembrane | 165 | 185 | 21 |
| GsPIP2;10 | Transmembrane | 124 | 144 | 21 |

| | | | | |
|---|---|---|---|---|
| GsPIP2;10 | Transmembrane | 248 | 268 | 21 |
| GsPIP2;10 | Transmembrane | 72 | 92 | 21 |
| GsPIP2;10 | Transmembrane | 198 | 218 | 21 |
| GsPIP2;10 | Transmembrane | 95 | 115 | 21 |
| GsPIP2;10 | Transmembrane | 39 | 59 | 21 |
| GsPIP2;10 | Transmembrane | 165 | 185 | 21 |
| GsPIP2;14 | Transmembrane | 122 | 143 | 22 |
| GsPIP2;14 | Transmembrane | 247 | 267 | 21 |
| GsPIP2;14 | Transmembrane | 81 | 101 | 21 |
| GsPIP2;14 | Transmembrane | 197 | 217 | 21 |
| GsPIP2;14 | Transmembrane | 40 | 60 | 21 |
| GsPIP2;14 | Transmembrane | 164 | 184 | 21 |
| GsPIP2;13 | Transmembrane | 122 | 142 | 21 |
| GsPIP2;13 | Transmembrane | 250 | 270 | 21 |
| GsPIP2;13 | Transmembrane | 81 | 101 | 21 |
| GsPIP2;13 | Transmembrane | 200 | 220 | 21 |
| GsPIP2;13 | Transmembrane | 40 | 60 | 21 |
| GsPIP2;13 | Transmembrane | 163 | 183 | 21 |
| GsPIP2;3 | Transmembrane | 123 | 143 | 21 |
| GsPIP2;3 | Transmembrane | 251 | 271 | 21 |
| GsPIP2;3 | Transmembrane | 202 | 222 | 21 |
| GsPIP2;3 | Transmembrane | 82 | 102 | 21 |
| GsPIP2;3 | Transmembrane | 169 | 189 | 21 |
| GsPIP2;3 | Transmembrane | 41 | 61 | 21 |
| GsPIP2;4 | Transmembrane | 123 | 143 | 21 |
| GsPIP2;4 | Transmembrane | 251 | 271 | 21 |
| GsPIP2;4 | Transmembrane | 202 | 222 | 21 |
| GsPIP2;4 | Transmembrane | 82 | 102 | 21 |
| GsPIP2;4 | Transmembrane | 169 | 189 | 21 |
| GsPIP2;4 | Transmembrane | 41 | 61 | 21 |
| GsPIP2;6 | Transmembrane | 124 | 144 | 21 |
| GsPIP2;6 | Transmembrane | 252 | 272 | 21 |
| GsPIP2;6 | Transmembrane | 203 | 223 | 21 |
| GsPIP2;6 | Transmembrane | 83 | 103 | 21 |
| GsPIP2;6 | Transmembrane | 170 | 190 | 21 |
| GsPIP2;6 | Transmembrane | 42 | 62 | 21 |
| GsPIP2;5 | Transmembrane | 124 | 144 | 21 |
| GsPIP2;5 | Transmembrane | 252 | 272 | 21 |
| GsPIP2;5 | Transmembrane | 203 | 223 | 21 |
| GsPIP2;5 | Transmembrane | 83 | 103 | 21 |
| GsPIP2;5 | Transmembrane | 170 | 190 | 21 |
| GsPIP2;5 | Transmembrane | 42 | 62 | 21 |
| GsTIP5;1 | Transmembrane | 59 | 79 | 21 |
| GsTIP5;1 | Transmembrane | 204 | 224 | 21 |
| GsTIP5;1 | Transmembrane | 82 | 109 | 28 |
| GsTIP5;1 | Transmembrane | 163 | 183 | 21 |

| | | | | |
|---|---|---|---|---|
| GsTIP5;1 | Transmembrane | 17 | 38 | 22 |
| GsTIP5;1 | Transmembrane | 130 | 150 | 21 |
| GsTIP3;1 | Transmembrane | 100 | 120 | 21 |
| GsTIP3;1 | Transmembrane | 218 | 239 | 22 |
| GsTIP3;1 | Transmembrane | 177 | 197 | 21 |
| GsTIP3;1 | Transmembrane | 59 | 79 | 21 |
| GsTIP3;1 | Transmembrane | 141 | 161 | 21 |
| GsTIP3;2 | Transmembrane | 100 | 120 | 21 |
| GsTIP3;2 | Transmembrane | 218 | 239 | 22 |
| GsTIP3;2 | Transmembrane | 177 | 197 | 21 |
| GsTIP3;2 | Transmembrane | 59 | 79 | 21 |
| GsTIP3;2 | Transmembrane | 141 | 161 | 21 |
| GsTIP3;3 | Transmembrane | 100 | 120 | 21 |
| GsTIP3;3 | Transmembrane | 214 | 234 | 21 |
| GsTIP3;3 | Transmembrane | 173 | 193 | 21 |
| GsTIP3;3 | Transmembrane | 59 | 79 | 21 |
| GsTIP3;3 | Transmembrane | 141 | 161 | 21 |
| GsTIP3;4 | Transmembrane | 100 | 120 | 21 |
| GsTIP3;4 | Transmembrane | 214 | 234 | 21 |
| GsTIP3;4 | Transmembrane | 173 | 193 | 21 |
| GsTIP3;4 | Transmembrane | 59 | 79 | 21 |
| GsTIP3;4 | Transmembrane | 141 | 161 | 21 |
| GsTIP4;2 | Transmembrane | 98 | 118 | 21 |
| GsTIP4;2 | Transmembrane | 213 | 233 | 21 |
| GsTIP4;2 | Transmembrane | 50 | 77 | 28 |
| GsTIP4;2 | Transmembrane | 172 | 192 | 21 |
| GsTIP4;2 | Transmembrane | 21 | 41 | 21 |
| GsTIP4;2 | Transmembrane | 139 | 159 | 21 |
| GsTIP4;1 | Transmembrane | 98 | 118 | 21 |
| GsTIP4;1 | Transmembrane | 213 | 233 | 21 |
| GsTIP4;1 | Transmembrane | 50 | 77 | 28 |
| GsTIP4;1 | Transmembrane | 172 | 192 | 21 |
| GsTIP4;1 | Transmembrane | 21 | 41 | 21 |
| GsTIP4;1 | Transmembrane | 139 | 159 | 21 |
| GsTIP1;4 | Transmembrane | 103 | 123 | 21 |
| GsTIP1;4 | Transmembrane | 214 | 234 | 21 |
| GsTIP1;4 | Transmembrane | 173 | 193 | 21 |
| GsTIP1;4 | Transmembrane | 62 | 82 | 21 |
| GsTIP1;4 | Transmembrane | 21 | 41 | 21 |
| GsTIP1;4 | Transmembrane | 144 | 164 | 21 |
| GsTIP1;5 | Transmembrane | 103 | 123 | 21 |
| GsTIP1;5 | Transmembrane | 214 | 234 | 21 |
| GsTIP1;5 | Transmembrane | 173 | 193 | 21 |
| GsTIP1;5 | Transmembrane | 62 | 82 | 21 |
| GsTIP1;5 | Transmembrane | 21 | 41 | 21 |
| GsTIP1;5 | Transmembrane | 144 | 164 | 21 |

| | | | | |
|---|---|---|---|---|
| GsTIP1;6 | Transmembrane | 103 | 123 | 21 |
| GsTIP1;6 | Transmembrane | 219 | 239 | 21 |
| GsTIP1;6 | Transmembrane | 173 | 193 | 21 |
| GsTIP1;6 | Transmembrane | 62 | 82 | 21 |
| GsTIP1;6 | Transmembrane | 21 | 41 | 21 |
| GsTIP1;6 | Transmembrane | 144 | 164 | 21 |
| GsTIP1;7 | Transmembrane | 62 | 82 | 21 |
| GsTIP1;7 | Transmembrane | 132 | 152 | 21 |
| GsTIP1;7 | Transmembrane | 21 | 41 | 21 |
| GsTIP1;7 | Transmembrane | 103 | 123 | 21 |
| GsTIP1;3 | Transmembrane | 104 | 124 | 21 |
| GsTIP1;3 | Transmembrane | 17 | 37 | 21 |
| GsTIP1;3 | Transmembrane | 214 | 234 | 21 |
| GsTIP1;3 | Transmembrane | 173 | 193 | 21 |
| GsTIP1;3 | Transmembrane | 58 | 78 | 21 |
| GsTIP1;3 | Transmembrane | 81 | 101 | 21 |
| GsTIP1;3 | Transmembrane | 141 | 161 | 21 |
| GsTIP1;1 | Transmembrane | 103 | 123 | 21 |
| GsTIP1;1 | Transmembrane | 214 | 234 | 21 |
| GsTIP1;1 | Transmembrane | 173 | 193 | 21 |
| GsTIP1;1 | Transmembrane | 62 | 82 | 21 |
| GsTIP1;1 | Transmembrane | 21 | 41 | 21 |
| GsTIP1;1 | Transmembrane | 144 | 164 | 21 |
| GsTIP1;2 | Transmembrane | 103 | 123 | 21 |
| GsTIP1;2 | Transmembrane | 214 | 234 | 21 |
| GsTIP1;2 | Transmembrane | 173 | 193 | 21 |
| GsTIP1;2 | Transmembrane | 62 | 82 | 21 |
| GsTIP1;2 | Transmembrane | 21 | 41 | 21 |
| GsTIP1;2 | Transmembrane | 144 | 164 | 21 |
| GsTIP2;1 | Transmembrane | 109 | 128 | 20 |
| GsTIP2;1 | Transmembrane | 211 | 231 | 21 |
| GsTIP2;1 | Transmembrane | 170 | 190 | 21 |
| GsTIP2;1 | Transmembrane | 57 | 77 | 21 |
| GsTIP2;1 | Transmembrane | 86 | 106 | 21 |
| GsTIP2;1 | Transmembrane | 21 | 41 | 21 |
| GsTIP2;1 | Transmembrane | 141 | 161 | 21 |
| GsTIP2;2 | Transmembrane | 109 | 128 | 20 |
| GsTIP2;2 | Transmembrane | 211 | 231 | 21 |
| GsTIP2;2 | Transmembrane | 170 | 190 | 21 |
| GsTIP2;2 | Transmembrane | 57 | 77 | 21 |
| GsTIP2;2 | Transmembrane | 86 | 106 | 21 |
| GsTIP2;2 | Transmembrane | 21 | 41 | 21 |
| GsTIP2;2 | Transmembrane | 141 | 161 | 21 |
| GsTIP2;4 | Transmembrane | 102 | 122 | 21 |
| GsTIP2;4 | Transmembrane | 216 | 236 | 21 |
| GsTIP2;4 | Transmembrane | 175 | 195 | 21 |

| | | | | |
|---|---|---|---|---|
| GsTIP2;4 | Transmembrane | 54 | 74 | 21 |
| GsTIP2;4 | Transmembrane | 79 | 99 | 21 |
| GsTIP2;4 | Transmembrane | 21 | 41 | 21 |
| GsTIP2;4 | Transmembrane | 134 | 154 | 21 |
| GsTIP2;3 | Transmembrane | 102 | 122 | 21 |
| GsTIP2;3 | Transmembrane | 216 | 236 | 21 |
| GsTIP2;3 | Transmembrane | 175 | 195 | 21 |
| GsTIP2;3 | Transmembrane | 54 | 74 | 21 |
| GsTIP2;3 | Transmembrane | 79 | 99 | 21 |
| GsTIP2;3 | Transmembrane | 21 | 41 | 21 |
| GsTIP2;3 | Transmembrane | 134 | 154 | 21 |
| GsTIP2;7 | Transmembrane | 99 | 119 | 21 |
| GsTIP2;7 | Transmembrane | 215 | 235 | 21 |
| GsTIP2;7 | Transmembrane | 174 | 194 | 21 |
| GsTIP2;7 | Transmembrane | 58 | 78 | 21 |
| GsTIP2;7 | Transmembrane | 132 | 153 | 22 |
| GsTIP2;7 | Transmembrane | 21 | 41 | 21 |
| GsTIP2;6 | Transmembrane | 99 | 119 | 21 |
| GsTIP2;6 | Transmembrane | 215 | 235 | 21 |
| GsTIP2;6 | Transmembrane | 174 | 194 | 21 |
| GsTIP2;6 | Transmembrane | 58 | 78 | 21 |
| GsTIP2;6 | Transmembrane | 132 | 153 | 22 |
| GsTIP2;6 | Transmembrane | 21 | 41 | 21 |
| GsTIP2;5 | Transmembrane | 99 | 119 | 21 |
| GsTIP2;5 | Transmembrane | 215 | 235 | 21 |
| GsTIP2;5 | Transmembrane | 174 | 194 | 21 |
| GsTIP2;5 | Transmembrane | 58 | 78 | 21 |
| GsTIP2;5 | Transmembrane | 132 | 153 | 22 |
| GsTIP2;5 | Transmembrane | 21 | 41 | 21 |
| GsSIP2;1 | Transmembrane | 200 | 220 | 21 |
| GsSIP2;1 | Transmembrane | 13 | 34 | 22 |
| GsSIP2;1 | Transmembrane | 167 | 187 | 21 |
| GsSIP2;1 | Transmembrane | 39 | 59 | 21 |
| GsSIP2;1 | Transmembrane | 126 | 146 | 21 |
| GsSIP1;6 | Transmembrane | 206 | 226 | 21 |
| GsSIP1;6 | Transmembrane | 17 | 37 | 21 |
| GsSIP1;6 | Transmembrane | 165 | 185 | 21 |
| GsSIP1;6 | Transmembrane | 42 | 62 | 21 |
| GsSIP1;5 | Transmembrane | 210 | 230 | 21 |
| GsSIP1;5 | Transmembrane | 17 | 37 | 21 |
| GsSIP1;5 | Transmembrane | 162 | 182 | 21 |
| GsSIP1;5 | Transmembrane | 40 | 60 | 21 |
| GsSIP1;4 | Transmembrane | 95 | 116 | 22 |
| GsSIP1;4 | Transmembrane | 212 | 231 | 20 |
| GsSIP1;4 | Transmembrane | 171 | 191 | 21 |
| GsSIP1;4 | Transmembrane | 137 | 158 | 22 |

| | | | | |
|---|---|---|---|---|
| GsSIP1;4 | Transmembrane | 13 | 33 | 21 |
| GsSIP1;4 | Transmembrane | 54 | 74 | 21 |
| GsSIP1;3 | Transmembrane | 95 | 116 | 22 |
| GsSIP1;3 | Transmembrane | 212 | 231 | 20 |
| GsSIP1;3 | Transmembrane | 171 | 191 | 21 |
| GsSIP1;3 | Transmembrane | 137 | 158 | 22 |
| GsSIP1;3 | Transmembrane | 13 | 33 | 21 |
| GsSIP1;3 | Transmembrane | 54 | 74 | 21 |
| GsSIP1;2 | Transmembrane | 95 | 116 | 22 |
| GsSIP1;2 | Transmembrane | 213 | 234 | 22 |
| GsSIP1;2 | Transmembrane | 171 | 192 | 22 |
| GsSIP1;2 | Transmembrane | 137 | 158 | 22 |
| GsSIP1;2 | Transmembrane | 13 | 33 | 21 |
| GsSIP1;2 | Transmembrane | 54 | 74 | 21 |
| GsSIP1;1 | Transmembrane | 50 | 77 | 28 |
| GsSIP1;1 | Transmembrane | 221 | 241 | 21 |
| GsSIP1;1 | Transmembrane | 9 | 29 | 21 |
| GsSIP1;1 | Transmembrane | 151 | 171 | 21 |
| GsSIP1;1 | Transmembrane | 192 | 212 | 21 |

Supplementary Table S2

The list of primers used for RT-PCR in this study.

| Gene name | Forward primer | Reverse primer |
|---|---|---|
| *GmTIP2;1* | TGTAGTAAGTGAGGTGCCAAT | AACCCAGTAGATCCAGTTAGC |
| *GmTIP1;7* | ATGCCGATCAGAAACATCGCCAT | CTAGTAGTCAGTGCTGGGAAGC |
| *GmTIP4;1* | TCTGCAATTGAGCAGAGGAGCC | CTCATAGATGTAACCAGCAAGG |
| *GmPIP2;3* | ACTTATTTGAGGTTGGAGAAAT | GCAGAGAAGACAGTGTAGACAA |
| *GmPIP1;4* | TTTCTTGAGTTGAGTTAACAGC | AGAGTCTCTAGCGTTTCTCTTG |
| *GmXIP1;1* | CCTCTCTAATGTTCACCTTGAC | CCCAGTTACAGTTATGGACAC |
| *GmTIP3;3* | AAGGTCTATTTGATCAGGGAT | GTATTCATACAGGAGTGCTGC |
| *GmTIP1;4* | GCTTATAGTTGAAATCTTTGAG | CCCAGTAGACCCAGTGATTA |
| *GmPIP2;4* | AGCACACAAAACTTGCTATAG | GCAGAGAAGACAGTGTAGACAA |
| *GmPIP1;8* | AGAAATAGCTAGAGTGAGAAAG | AATAGGGACGTGTGAGTCTCT |
| *GmTIP1;1* | ATGGCAATATATAGAATTGCAAT | CTAGAAATCACTGCTTGAAAGAG |
| *GmXIP1;2* | TCAACTCAAATAGTTGTTGGT | CTCCATCACACTCTTGAGTACA |
| *GmSIP1;5* | ATGGTTAGTGCAATAAAAGCAGCC | TCAAGCTTTCTTCTGCTTGATTGG |
| *GmPIP2;6* | GGAATCTGCTAGAATTTTCTAAT | CTAACACAGGAACATGAGAGTC |
| *GmTIP1;9* | GTCTGATCTCGATCGCACGGAC | GGCTATGGCTAACAAAGATG |
| *GmNIP6;2* | ATAAGGGTAGTTGCAAATTGCA | GCCCTGCTATGAGTATATTGAG |
| *GmTIP2;6* | TTCTCACGGAAACTTCTGAAG | GGGATGAAGGCATAGGTATAG |
| *GmPIP2;11* | TAAGGGTAGTTGCAAATTGCA | TAGACTCCTAGCAGGGTTAATG |
| *GmPIP2;10* | ACTCACACAGTGGTCCTTCTAC | CAGAGAACACAGTGTAGACCAA |
| *GmTIP1;2* | GTCTCATTGCTGCATCACTATC | ACCAACTAAGATGTTGGCAC |
| *GmTIP1;8* | CATAAGGGTAGTTGCAAATTGC | CAGTAGATCCAGTGGTTGGT |
| *GmSIP1;3* | ATGGGGTTGATCAAAGCAGCAATTG | TCAAGCTTTCTTCTGCTTGATTGGT |
| *GmSIP1;1* | ATGGTTAGTGCAATAAAAGCAGCC | TCAGGCTTTCTTCTGCTTTACCAC |
| *GmSIP1;2* | ATGGCTAGTGCTATAAAAGCTGCC | TCAGGCTTTCTTCTGCTTTACCA |
| *GmPIP1;7* | AGAATCCCAGGCGAGGAGAAAG | ATGATAGCAGCACCAAGACTAC |
| Q*GmTIP2;1* | GTGAAGATAGCTCTTGGT | TCAAGGGTAGGTTTCAGAGGCTGG |
| Q*GmTIP1;7* | CCGATCAGAAACATCGCC | TAGTAGTCAGTGCTGGGAAGCTG |
| Q*GmTIP1;8* | ATGCCGATCAGAAACATCGCC | CTAGTAGTCAGTGGTGGGAAG |

Supplementary Table S3

The list of primers used for the yeast and BiFC experiments.

| Gene name | Forward primer | Reverse primer |
|---|---|---|
| Y2H*GmTIP2;1* | cgCCATGGATGGTGAAGATAGCTCTTGGT | cGGAATTCTCAAGGGTAGGTTTCAGAGGCTGG |
| Y2H*GmTIP1;7* | cgCCATGGATGCCGATCAGAAACATCGCC | cGGAATTCCTAGTAGTCAGTGCTGGGAAGCTG |
| Y2H*GmTIP1;8* | cgCCATGGATGCCGATCAGAAACATCGCC | cGGAATTCCTAGTAGTCAGTGGTGGGAAG |
| Y*GmTIP2;1* | cgAAGCTT ATGGTGAAGATAGCTCTTGGT | cGTCTAGAAGGGTAGGTTTCAGAGGCTGG |
| YGmTIP1;7 | cgAAGCTTATGCCGATCAGAAACATCGCC | cGTCTAGAGTAGTCAGTGCTGGGAAGCTG |
| Y*GmTIP1;8* | cgAAGCTTATGCCGATCAGAAACATCGCC | cGTCTAGAGTAGTCAGTGGTGGGAAG |
| *Y2HPip2;3* | cgCCATGGATGGCTAAAGATGTTGAGGT | cGGAATTCTTAAGCGTTGCTTCTGAAGGA |
| *Y2HPip1;4* | cgCCATGGATGGAGAGGGAGGAAGATGT | cGGAATTCTTAACCCCTTGTCTTGAATGG |
| *Y2HPip2;4* | cgCCATGGATGGCTAAAGATGTTGAGGTT | cGGAATTCTTAAGCGTTGCTCCTGAAGGA |
| *Y2HPip1;8* | cgCCATGGATGGCTAAAGATGTTGAGGTT | cGGAATTCTTAACTGGATTTGAAGGGAAT |
| *Y2HPip2;6* | cgCCATGGATGGCGAAAGACGTTGAGCA | cGGAATTCTTAAGCGTTGCTCCTGAAGGA |
| *Y2HPip2;10* | cgCCATGGATGGCTAAGCATGATGTTGAG | cGGAATTCTCAAATAGCGGGGTTGCTCCT |
| *Y2HPip2;11* | cgCCATGGATGGCTAAGCATGATGTTGAG | cGGAATTCTCAAATAGTGGGGTTGCTCCT |
| *Y2HPip1;7* | cgCCATGGATGGAGGGGAAGGAGCAGGA | cGGAATTCTCACTTGGACTTGAAGGGAAT |
| *BiFCGmTIP2;1* | cgCTCGAGATGGTGAAGATAGCTCTTGGT | cGCCCGGGAGGGTAGGTTTCAGAGGCTGG |
| *BiFCGmTIP1;7* | cgCTCGAGATGCCGATCAGAAACATCGCC | cGCCCGGGGTAGTCAGTGCTGGGAAGCTG |
| *BiFCGmTIP1;8* | cgCTCGAGATGCCGATCAGAAACATCGCC | cGCCCGGGGTAGTCAGTGGTGGGAAG |
